# Supplementary material for: MyD88 TIR domain higher-order assembly interactions revealed by microcrystal electron diffraction and serial femtosecond crystallography
Source: Nat Commun. 2021 May 10;12:2578. doi: 10.1038/s41467-021-22590-6 (PMC8110528; doi:10.1038/s41467-021-22590-6)
Supplement: Supplementary file 1 — Supplementary Information [file 41467_2021_22590_MOESM1_ESM.pdf]

Supplementary Information

MyD88 TIR domain higher-order assembly interactions revealed by microcrystal electron diffraction and serial femtosecond crystallography

Clabbers *et al.*

Supplementary Figures

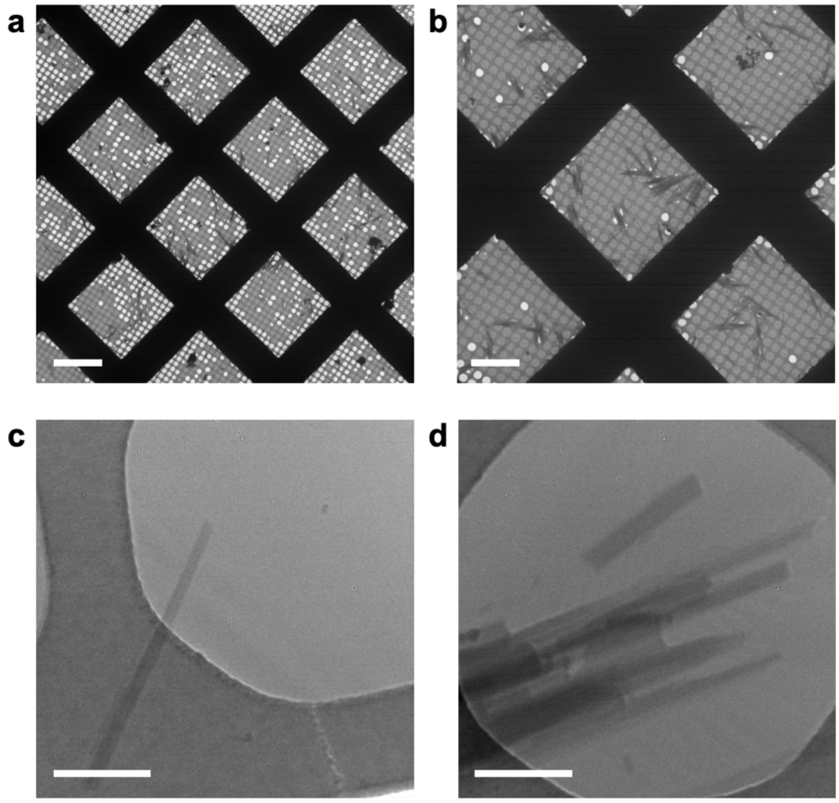

Supplementary Figure 1. Micrographs of MyD88^{TIR} higher-order assembly microcrystals at different magnifications. a. Grid overview showing even distribution of microcrystals. Scale bar, 30 μm. **b.** Grid squares show substantial aggregation of microcrystals into bundles. Scale bar, 15 μm. **c.** Single hydrated microcrystal over a hole in the carbon support layer. Scale bar, 1 μm. **d.** Bundle of hydrated MyD88^{TIR} microcrystals. Scale bar, 1 μm. The data in **a-d** are representative of three EM grids prepared using 3μl of a 1:50 MAL^{TIR}:MyD88^{TIR} crystal solution.

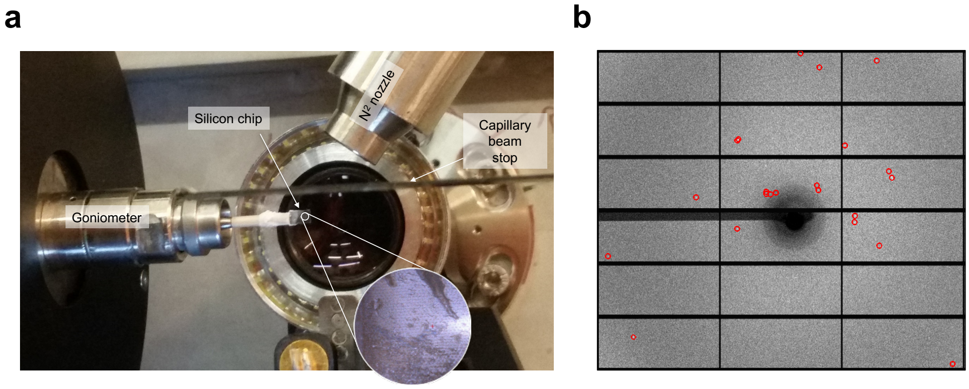

Supplementary Figure 2. **Crystallography set-up at PETRA III using the static chip system. a.** MyD88^{TIR} sample set-up at the P11 beam-line at the Petra III synchrotron. The sample was placed on a silicon chip which was subsequently frozen in liquid nitrogen and mounted on the goniometer under the cryogenic nitrogen stream. The insert shows a close-up image of microcrystal aggregation on the silicon chip with a crystal concentration of 3.3x10^8 crystals/ml. This led to multiple crystal diffraction patterns in individual images, which could not be reliably indexed. **b.** A diffraction image obtained via the chip method at the P11 beam-line. Red circles highlight Bragg peaks identified via CrystFEL.

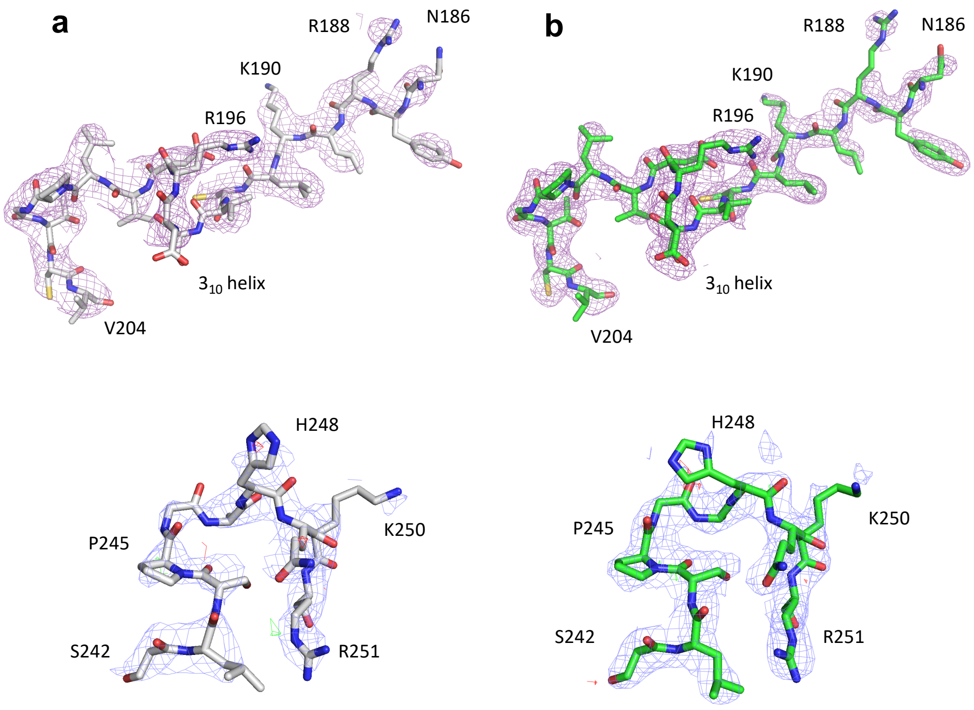

Supplementary Figure 3. Model building and refinement of MyD88^{TIR}. Models and simulated annealing (SA) composite omit maps (contoured at 1.0σ) are presented for the **a.** MicroED and **b.** SFX[b] structures. Both structures were solved, built and refined using an identical protocol to enable a direct comparison. The BB loop (magenta isomesh, residues 186-204; top) and CD loop (blue isomesh, residues 242-251; bottom) regions are shown. The carbon atoms in the MicroED and SFX structures are shown in grey and green, respectively. Nitrogen, oxygen and sulfur atoms are shown in blue, red and yellow, respectively. The SA composite omit maps were calculated over the entire unit cell.

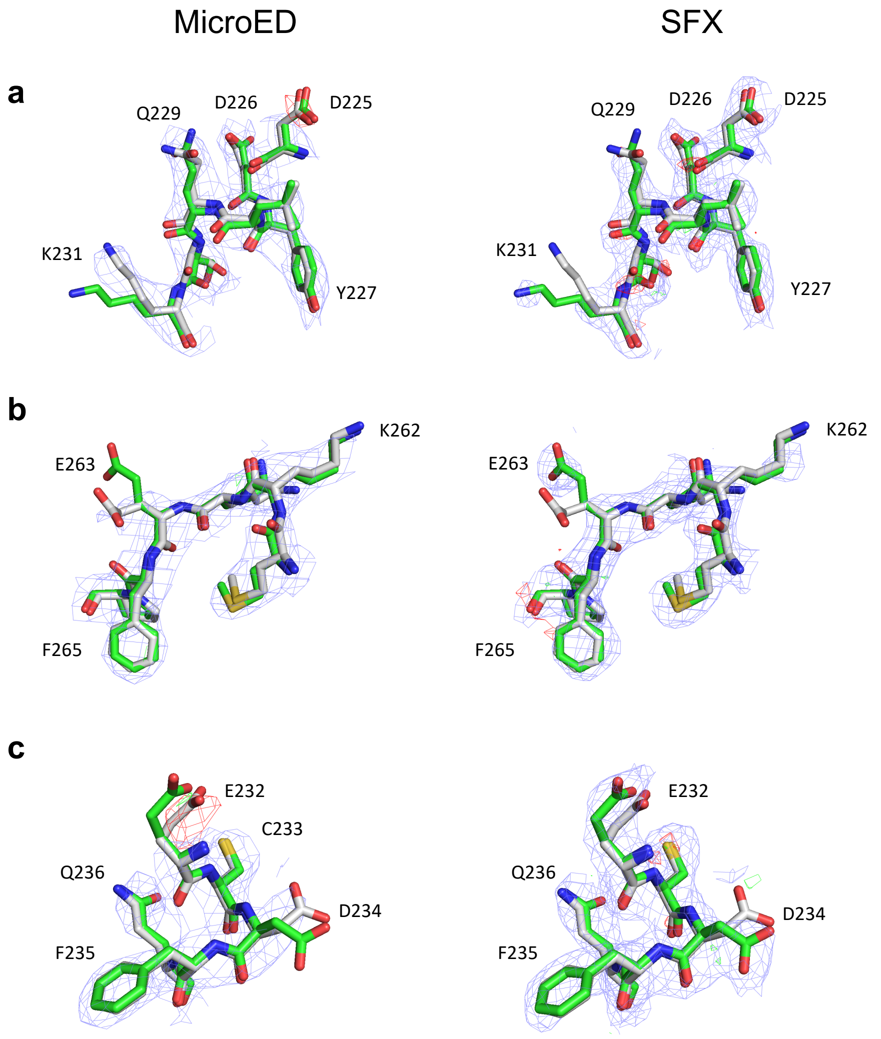

Supplementary Figure 4. Structural comparison between the MicroED and SFX derived structures. The aligned MicroED (grey) and SFX[b] (green) models are shown with electrostatic potential (MicroED; left panel) and electron density (SFX[b]; right panel) maps. The models and maps are presented at four different sites; **a** D225-K231, **b** K261-P265, and **c** E232-Q236. The structures were solved, built and refined using an identical protocol to enable a direct comparison between the two methods. The carbon atoms in the MicroED and SFX structures are shown in grey and green, respectively. Nitrogen, oxygen and sulfur atoms are shown in blue, red and yellow, respectively. Electrostatic potential and electron density 2mFo-DFc maps (blue isomesh) are contoured at 1.0σ, and the difference mFo-DFc maps (green and red isomesh, for positive and negative density, respectively) are contoured at 3.0σ. No missing reflections were restored using weighted Fc values for map calculations.

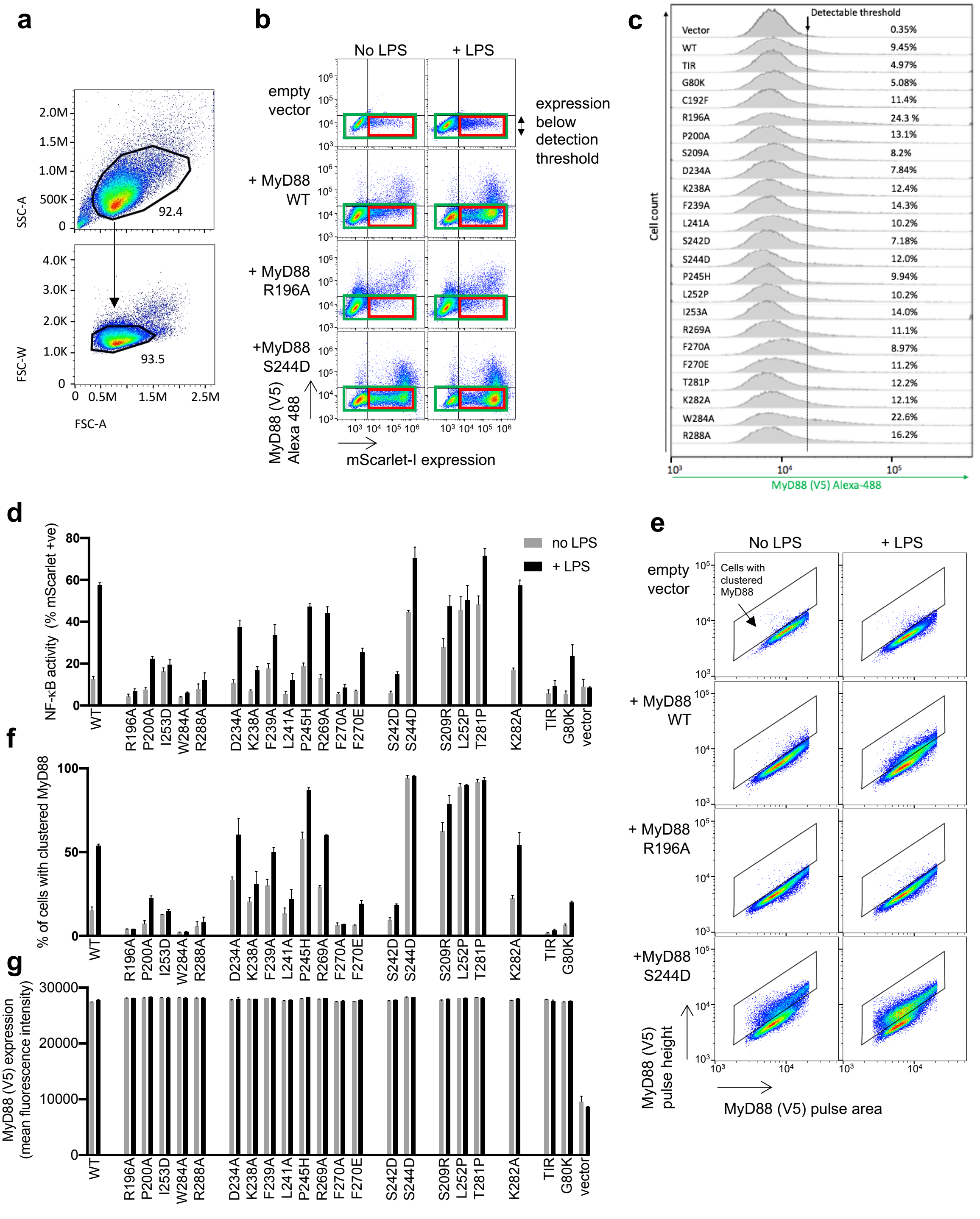

Supplementary Figure 5. Details of the flow cytometry-based assay for evaluation of MyD88 mutants. A HEK293 reporter cell line was prepared that stably expresses human TLR4, MD-2 and CD14, with an integrated NF-κB-driven mScarlet-I reporter plasmid, and endogenous *MYD88* knocked out. The resulting cell line (HEK-Blue-TLR4-NF-κB-mScar-MyD88 KO) was transfected with plasmids expressing V5-tagged wild-type or mutant MyD88 and then treated with or without 100 ng/ml LPS. The amount of plasmid used was titrated down to give minimal spontaneous signaling. **a**. Gating on side scatter (SSC) vs forward scatter (FSC) plot of all events collected, to eliminate debris from intact cell population (upper panel) and then on FSC-width vs FSC-area plot of the intact cell population to obtain a single-cell

population (lower panel) used for all subsequent flow cytometry analysis. **b.** Reconstitution of normal LPS-inducible NF-κB signaling by WT but not mutant MyD88. Plot of MyD88-V5 expression vs mScarlet-I expression for selected samples with or without LPS treatment. Some basal mScarlet-I expression is seen in cells transfected with empty vector, and is at least partly related to transfection stress. Cells with WT MyD88 levels that were not above the background fluorescence level seen with empty vector transfection, still show induction of mScarlet-I expression upon stimulation with LPS. Because the cells are strongly responding to LPS stimulation we surmise that levels of MyD88 below the detection threshold are sufficient for function. This very low level of expression was selected for analysis in Figure 6a-b, as higher levels show spontaneous signaling. Defective mutants, such as R196A, do not respond to LPS at this MyD88 expression level. By contrast, constitutively active mutants, such as S244D, show elevated mScarlet expression in the absence of LPS. The geometric mean fluorescence signal within the red window was used for analysis of signaling response in Fig. 6a, to avoid including potentially variable numbers of untransfected cells. **c.** All MyD88 mutants can be expressed at a level above the signal for the empty vector. The total single cell population, gated as in panel a, is viewed as histograms for MyD88-V5 expression. **d.** NF-κB activation expressed as the percentage of mScarlet-positive cells within the green boxed window in panel a shows similar effects of mutations to Fig. 6a. **e.** MyD88 clustering in Fig. 6b was determined from fluorescence peak height-vs-area plots for MyD88-V5 signal from cells with low MyD88 within the green box gate in panel a. In cells with clustered MyD88, the peak height-to-area ratio of the MyD88-V5 signal is elevated, compared to that in cells with diffuse MyD88 localisation. Data for Fig. 6b were obtained from the percentage of cells with clustered MyD88 i.e. those falling within the marked gate. **f.** and **g.** MyD88 clustering was also determined at concentrations slightly above the MyD88 detection threshold. Panel f shows that at the mean expression level shown in panel g, there was a similar trend to that obtained at levels below the detection threshold (Fig. 6b), but greater saturation of the response. In d, f, and g, cells were treated with (black bars) or without (grey bars) 100 ng/ml LPS, and the mean +/- range from n=2 independent experiments are shown.

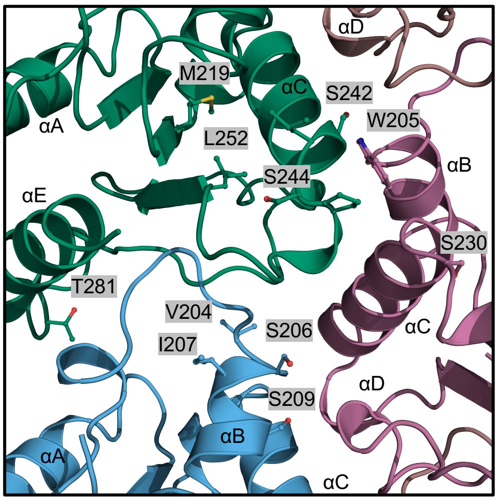

Supplementary Figure 6. Disease-associated and phosphomimetic mutations modulate MyD88 signaling and assembly. Ribbon diagram of the MyD88^{TIR} higher-order assembly with disease-associated and phosphorylated mutations highlighted in stick representation. The two strands of the higher-order assembly are shown in blue and green, and magenta and dark salmon, respectively.

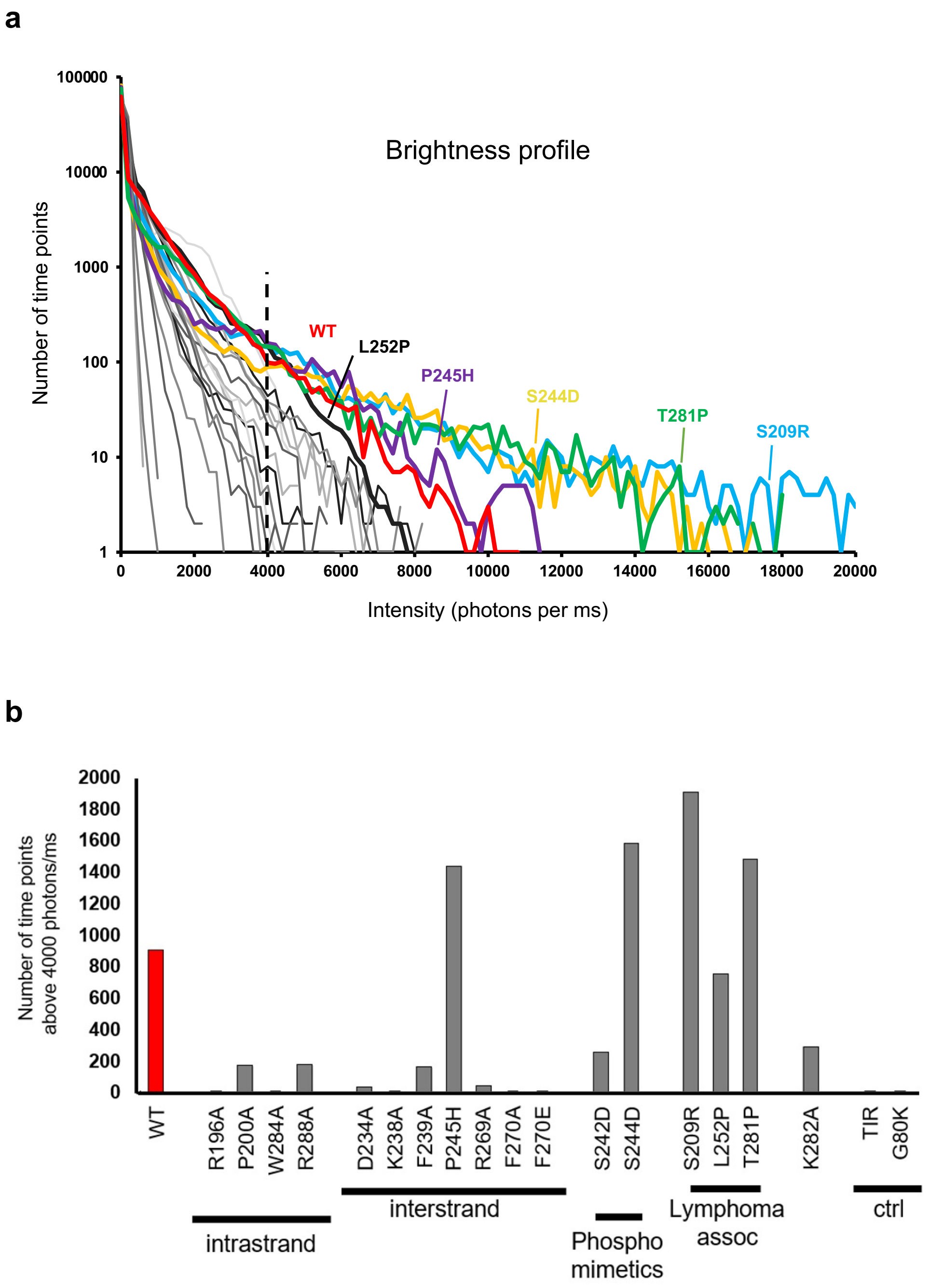

Supplementary Figure 7. Single-molecule analyses of MyD88^{TIR} assembly formation. a. Brightness profiles of MyD88 mutants**.** The brightness value in Fig. 6c represents the averaged value of many different particles. In inhomogeneous mixtures the brightness value alone cannot distinguish between rare, large particles or more frequent, but smaller particles. The maximum sizes of the assemblies were therefore analysed in more detail using the photon counting histogram (PCH) method[72] where the frequency of observed events are plotted as a function of their amplitude. Smaller particles yield many peaks at lower intensities while for larger particles the distribution is skewed towards higher intensities. In the case of MyD88, the PCH analysis show that only the S209R, S244D, P245H, and T281P form larger particles than wild-type MyD88. **b.** The ability of wild-type MyD88 and mutants to form large particles was also assessed by counting the number of events with high intensities. When the number of events above an arbitrary selection threshold of 4,000 photons/ms were plotted, the results clearly showed that the S209R, S244D, P245H and T281P mutants form larger particles than wild-type MyD88. Data in **b** show results from a single experiment (n=1).

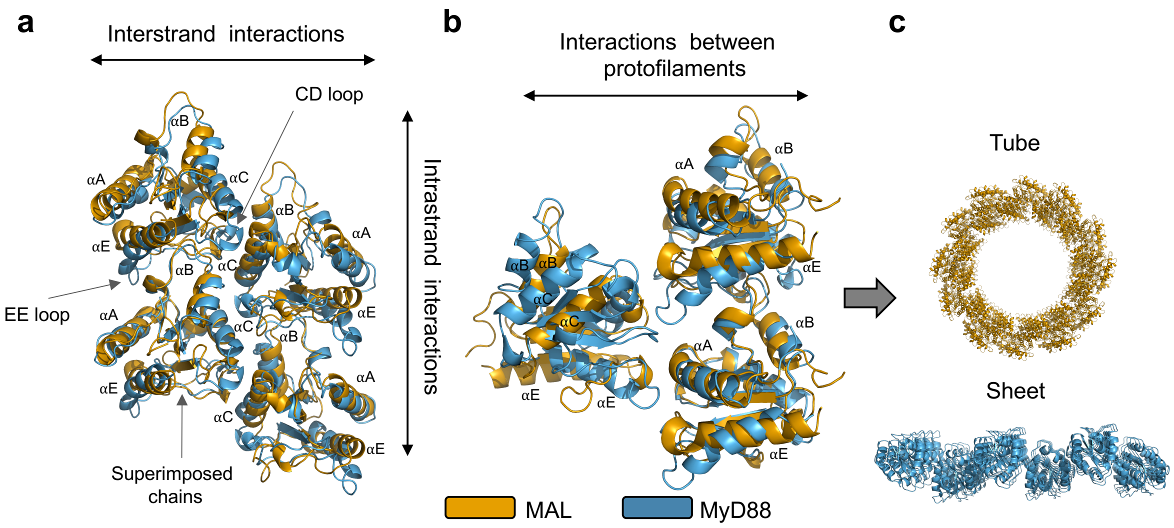

Supplementary Figure 8. Structural comparison of the MyD88^{TIR} microcrystal and MAL^{TIR} filament. a. Ribbon diagram comparing the MyD88^{TIR} (blue) and MAL^{TIR} (orange) higher-order assemblies. **b.** Ribbon diagram comparing the interactions between two-stranded higher-order assemblies in the MyD88^{TIR} microcrystal (blue) and MAL^{TIR} filament (orange). **c.** Arrangement of protofilaments in the MAL^{TIR} filaments (orange) and two-stranded assemblies in MyD88^{TIR} microcrystals (blue).

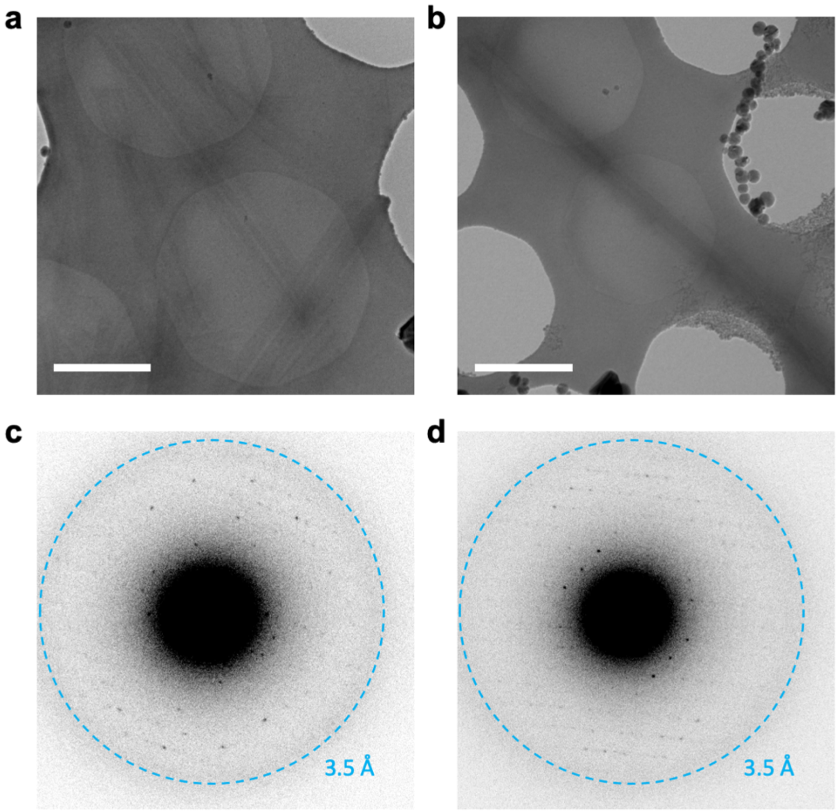

Supplementary Figure 9. Characterisation of GFP-MAL^{TIR} induced MyD88^{TIR} microcrystals by MicroED. a,b. Representative micrographs showing frozen hydrated GFP-MAL^{TIR}-seeded microcrystals on an EM grid. Scale bars: **a** 2 µm; **b** 1.5 µm. **c, d**. Representative electron diffraction patterns of GFP-MAL^{TIR}-seeded MyD88^{TIR} microcrystals, showing Bragg spots to 3.0 Å resolution. Diffraction patterns were collected with an angular increment of 0.68°, and a dose rate of 0.12 e^{-}/Å^{2} per frame. The diffraction patterns were indexed in space group *C*2 with unit cell parameters *a* = 101.077, *b* = 30,635, *c* = 57.713, and *β* = 110.326°. The data in **a-d** are representative of two EM grids prepared using 3µl of a 1:50 GFP-MAL^{TIR}:MyD88^{TIR} crystal solution.

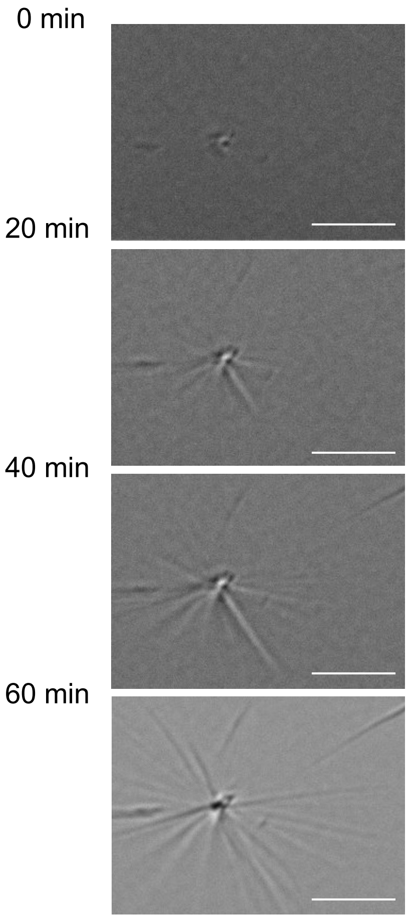

Supplementary Figure 10. Clustered microcrystals complicate analysis of unidirectional growth of MAL^{TIR} nucleated MyD88^{TIR} assembly. Time-lapse imaging of MyD88^{TIR} microcrystal formation. Representative images of multiple microcrystals, growing from a cluster of MAL^{TIR}-MyD88^{TIR} seeds, are shown. Data are representative of five independent experiments. Scale bar: 10 µm.

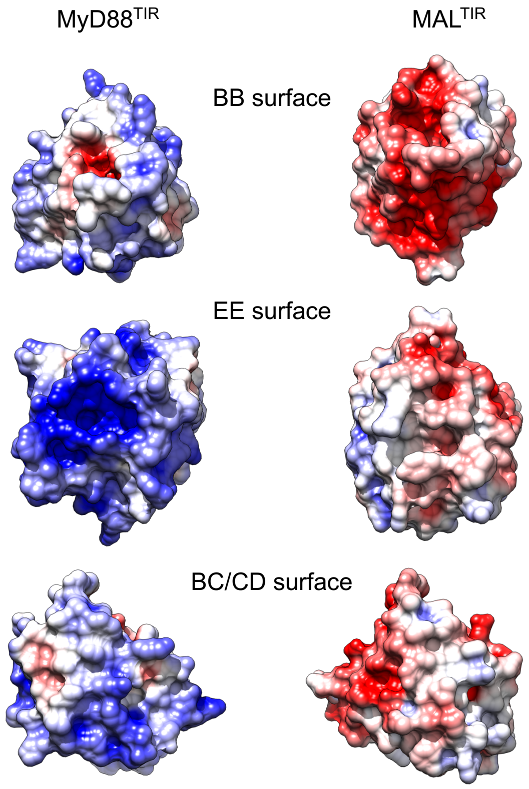

Supplementary Figure 11. Surface representations of MyD88^{TIR} and MAL^{TIR} with electrostatic potential mapped to the surface. Electrostatic potentials were calculated using APBS. Coloring is continuous going from blue (potential +10 kT/e) through white to red (potential −10 kT/e).

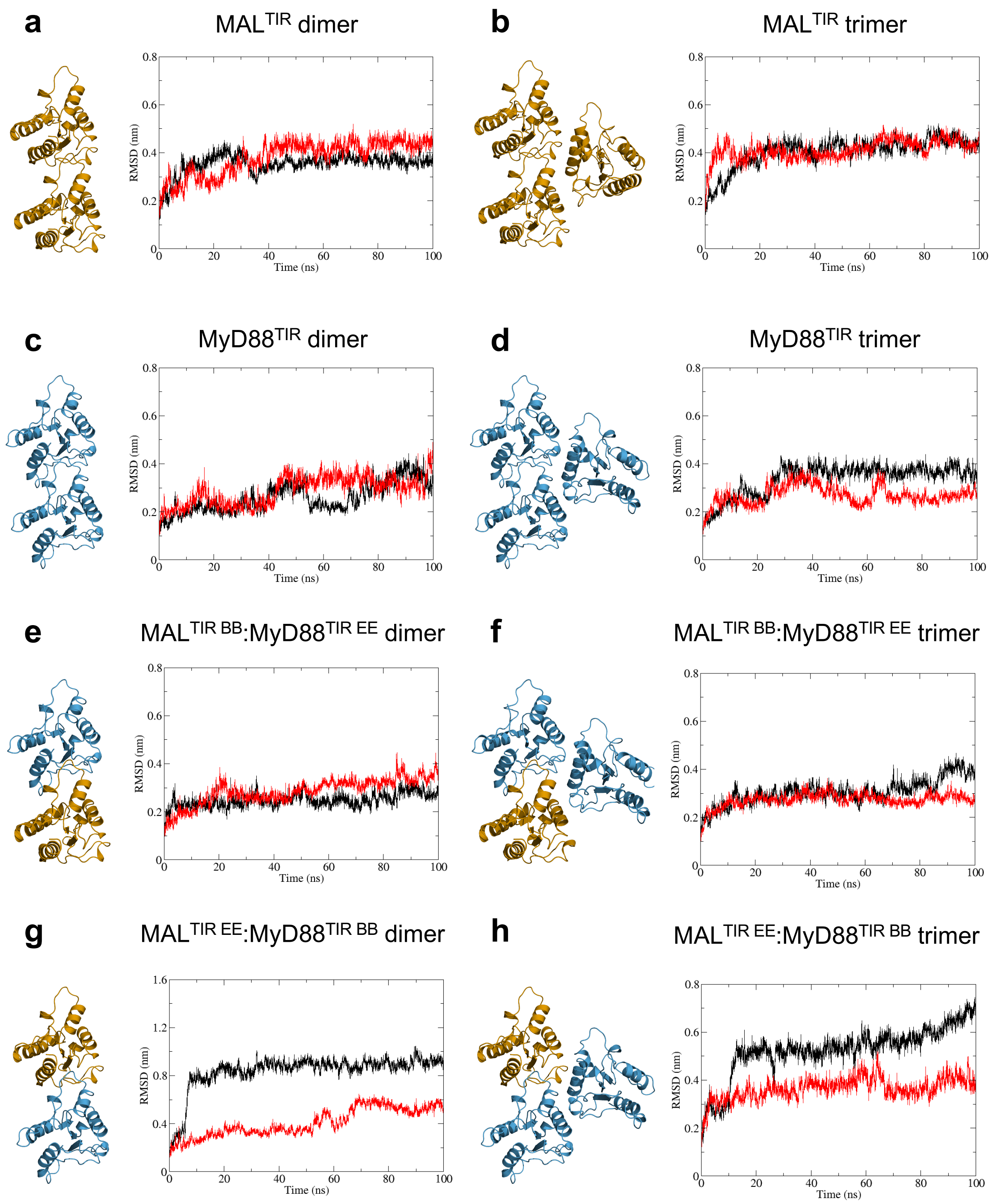

Supplementary Figure 12. Molecular-dynamics simulations of MyD88^{TIR} and MAL^{TIR} complexes. Starting structures and atom-positional RMSDs (root-mean-square deviation) of the backbone atoms (C_α, N, C) from duplicate 100 ns molecular dynamics simulations (black and red) of dimeric and trimeric MAL^{TIR} (**a-b**) and MyD88^{TIR} (**c-d**) complexes, and predicted MAL^{TIR} and MyD88^{TIR} complexes (**e-h**) using a box with a 1.4 nm distance between the protein surface and the edge of the box wall. The complexes analysed are highlighted in ribbon representation (MAL^{TIR} in orange; MyD88^{TIR} in blue). BB = BB surface of intrastrand interaction; EE = EE surface of intrastrand interaction. The RMSD analysis revealed that the complexes involving the MAL BB surface and MyD88 EE surface (**e-f**) are more stable than complexes involving the MAL EE surface and MyD88 BB surface (**g-h**).

Supplementary Table 1. **MyD88^{TIR} residues in SFX[a] structure contributing to the intrastrand interface as defined by PISA.**

Interface residues	Hydrogen bond (HB); salt bridge (SB) interactions
P169	-
I172	-
Q173	-
Q176	-
I179	-
V193	-
S194	-
D195	-
R196	-
V198	HB (R288)
L199	-
P200	-
G201	-
T202	HB (T272)
C203	-
V204	-
W205	-
S206	-
I207	-
G246	-
A247	-
H248	-
K250	-
R251	-
L252	-
I253	-
P254	-
R269	-
F270	-
I271	-
T272	HB (T202)
C274	-
N278	-
C280	-
T281	-
W284	-
R288	HB (V198)
L289	-
K291	-
A292	-
L295	-

Supplementary Table 2. **MyD88^{TIR} residues in SFX[a] structure contributing to the interstrand interface as defined by PISA.**

Interface residues	Hydrogen bond (HB); salt bridge (SB) interactions
C203	-
W205	HB (S242)
S206	-
S209	-
E213	SB (R269)
K231	-
E232	-
D234	SB (248)
F235	-
K238	-
F239	-
L241	-
S242	HB (W205)
L243	-
S244	-
P245	-
G246	-
H248	SB (D234)
S266	-
I267	-
R269	SB (E213)
F270	-

Supplementary Table 3. **MyD88^{TIR} residues in SFX[a] structure contributing to the interface between the two-stranded higher-order assemblies as defined by PISA.**

Interface residues	Hydrogen bond (HB); salt bridge (SB) interactions
S170	HB (K282)
I172	-
Q173	HB (Y276)
F174	-
Q176	-
E177	-
R180	-
Y257	-
K258	-
A259	-
Y276	HB (Q173)
T277	-
N278	-
P279	-
C280	-
K282	HB (S170)
S283	-

Supplementary Table 4. Comparison of the buried surface areas of different interfaces.

Interface	Buried surface area (Å^2)
MAL BB surface: MAL EE surface	1116.2
MyD88 BB surface: MyD88 EE surface[1]	1415.6
MAL BB surface: MyD88 EE surface (predicted)[1]	1576.4
MyD88 BB surface: MAL EE surface (predicted)[1]	1233.8
MAL BC surface: MAL CD surface	798.4
MyD88 BC surface: MyD88 CD surface[1]	965.2
MAL BC surface: MyD88 CD surface (predicted)[1]	764.4
MyD88 BC surface: MAL CD surface (predicted)[1]	934.0

[1] The calculated buried surface area is based on the MicroED MyD88^{TIR} structure

Supplementary Table 5. Primers used in this study.

Primer name	Primer sequence	Primer use
EGFP fwd	TACTTCCAATCCAATGCGAGCAAG GGCGAGGAGCTGTTCACC	GFP-MAL^{TIR}. Adds overhang for cloning into pMCSG7 vector via ligation independent cloning
EGFP rev	GGCTCCGGTGGCAGCTCTTCCCGTT GGTCTAAGGACTACG	GFP-MAL^{TIR}. Adds overhang for overlap PCR
MAL^{TIR} fwd	GCTGCCACCGGAGCCCTTGTACAGC TCGTCCATGCCGAG	GFP-MAL^{TIR}. Adds overhang for overlap PCR
MAL^{TIR} rev	TTATCCACTTCCAATGTTAAGAGAG GGTCTGCAGGTAGCGC	GFP-MAL^{TIR}. Adds overhang for cloning into pMCSG7 vector via ligation independent cloning
mScarlet-EcoRI fwd	GGAAGCTCGAATTCCAGCTTGGCAT TCCGGTACTGTTGGTAAAATGGTGA GCAAGGGC	mScarlet. Adds restriction site (EcoRI – mScarlet) for cloning into reporter plasmid
mScarlet-EcoRV rev	CCACCTGATATCTCACTTGTACAGC TCGTC	mScarlet. Adds restriction site (mScarlet – EcoRV) for cloning into reporter plasmid
AttB MyD88 fwd	GGGGACAAGTTTGTACAAAAAAGC AGGCTTCATGGCTGCAGGAGGTC	MyD88. Adds AttB site for Gateway™ cloning into pCellFree G03 vector
AttB MyD88 rev	GGGGACCACTTTGTACAAGAAAGC TGGGTTGGGCAGGGACAAGGC	MyD88. Adds AttB site for Gateway™ cloning into pCellFree G03 vector
