# Supplementary material for: MyD88 TIR domain higher-order assembly interactions revealed by microcrystal electron diffraction and serial femtosecond crystallography
Source: Nat Commun. 2021 May 10;12:2578. doi: 10.1038/s41467-021-22590-6 (PMC8110528; doi:10.1038/s41467-021-22590-6)
Supplement: Supplementary file 3 — Description of Additional Supplementary Files [file 41467_2021_22590_MOESM3_ESM.pdf]

Description of Additional Supplementary Files

Supplementary Movie 1. MAL^{TIR} nucleates $MyD88^{TIR}$ assembly formation unidirectionally. Movie of $MyD88^{TIR}$ microcrystal formation from GFP-MAL^{TIR}-$MyD88^{TIR}$ seeds. Scale bar: 10 μm
