# Supplementary material for: MyD88 TIR domain higher-order assembly interactions revealed by microcrystal electron diffraction and serial femtosecond crystallography
Source: Nat Commun. 2021 May 10;12:2578. doi: 10.1038/s41467-021-22590-6 (PMC8110528; doi:10.1038/s41467-021-22590-6)
Supplement: Supplementary file 5 — Reporting Summary [file 41467_2021_22590_MOESM5_ESM.pdf]

nature research

Corresponding author(s): Thomas Ve, Hongyi Xu, Bostjan Kobe, Connie Darmanin

Last updated by author(s): Mar 9, 2021

Reporting Summary

Nature Research wishes to improve the reproducibility of the work that we publish. This form provides structure for consistency and transparency in reporting. For further information on Nature Research policies, see our Editorial Policies and the Editorial Policy Checklist.

Statistics

For all statistical analyses, confirm that the following items are present in the figure legend, table legend, main text, or Methods section.

n/a	Confirmed	
☐	☒	The exact sample size (n) for each experimental group/condition, given as a discrete number and unit of measurement
☐	☒	A statement on whether measurements were taken from distinct samples or whether the same sample was measured repeatedly
☒	☐	The statistical test(s) used AND whether they are one- or two-sided<br>*Only common tests should be described solely by name; describe more complex techniques in the Methods section.*
☒	☐	A description of all covariates tested
☒	☐	A description of any assumptions or corrections, such as tests of normality and adjustment for multiple comparisons
☐	☒	A full description of the statistical parameters including central tendency (e.g. means) or other basic estimates (e.g. regression coefficient) AND variation (e.g. standard deviation) or associated estimates of uncertainty (e.g. confidence intervals)
☒	☐	For null hypothesis testing, the test statistic (e.g. F, t, r) with confidence intervals, effect sizes, degrees of freedom and P value noted<br>*Give P values as exact values whenever suitable.*
☒	☐	For Bayesian analysis, information on the choice of priors and Markov chain Monte Carlo settings
☒	☐	For hierarchical and complex designs, identification of the appropriate level for tests and full reporting of outcomes
☒	☐	Estimates of effect sizes (e.g. Cohen's d, Pearson's r), indicating how they were calculated

Our web collection on statistics for biologists contains articles on many of the points above.

Software and code

Policy information about availability of computer code

Data collection	Instamatic (version 1.0.0), Cheetah
Data analysis	CrystFEL software suite (0.9.0+b5de2753) including MOSFLM, XGANDALF, DIRax and partialator; XDS (version Mar 15, 2019 BUILT=20190315); Phenix software suite (1.1.4) including phenix.refine, Molprobity, composite_omit, and phenix.table_one; CCP4 software suite (CCP4-7.1000), AIMLESS (0.7.4), ctruncate (1.17.29) Phaser (version 2.8.2), Sculptor (0.3.3), MrBUMP (version 2.0.1), REFMAC (5.8.0258), Coot (0.8.9.2 and 0.9), ISOLDE (version 1.0b3), Dali server (http://ekhidna2.biocenter.helsinki.fi/dali), PISA server (https://www.ebi.ac.uk/pdbe/pisa), PIC server (http://pic.mbu.iisc.ernet.in), PyMOL (version 2.2.3), FlowJo 10.6.0, Gromacs 2019.3 (Gromos54a7 force field, SPC water model, Berendsen Barostat & Thermostat, LINCS & SETTLE algorithms).

For manuscripts utilizing custom algorithms or software that are central to the research but not yet described in published literature, software must be made available to editors and reviewers. We strongly encourage code deposition in a community repository (e.g. GitHub). See the Nature Research guidelines for submitting code & software for further information.

Data

Policy information about availability of data

All manuscripts must include a data availability statement. This statement should provide the following information, where applicable:

- Accession codes, unique identifiers, or web links for publicly available datasets
- A list of figures that have associated raw data
- A description of any restrictions on data availability

Data supporting the findings of this manuscript are available from the corresponding authors upon reasonable request. A reporting summary for this article is available as a supplementary information file. The atomic coordinates and structure factors of the MicroED and high-resolution SFX models (SFXa and SFXb) have been deposited in the Protein Data Bank under accession codes 7BEQ (doi:10.2210/pdb7BEQ/pdb, 7L6W (doi:10.2210/pdb7L6W/pdb) and 7BER (doi:10.2210/

pdb7BER/pdb), respectively. Raw MicroED data are available from the SBGrid Data Bank (doi:10.15785/SBGRID/814). SFX data are available at CXIB.org (doi:10.11577/1767965). The source data underlying Fig. 6 and Supplementary Fig. 5d,f-g are provided as a source data file.

Field-specific reporting

Please select the one below that is the best fit for your research. If you are not sure, read the appropriate sections before making your selection.

- [x] Life sciences
- [ ] Behavioural & social sciences
- [ ] Ecological, evolutionary & environmental sciences

For a reference copy of the document with all sections, see nature.com/documents/nr-reporting-summary-flat.pdf

Life sciences study design

All studies must disclose on these points even when the disclosure is negative.

Sample size

No statistical methods were used to pre-determine sample size. Quantitative data in Figure 6 and Supplementary Figures 5 and 6 is the result of either 2 or 3 independent experiments. For flow cytometry the results for each treatment group come from analysis of at least 2000 individual cells within the gated population, so in a sense each experiment has an internal n>2000. Our extensive recent experience with fluorescent reporter assays for NF-kB activity shows that data is entirely reproducible, and whether an experiment is repeated 2,3 or 4 times makes no substantial difference to the relative activity of different mutants.

Single molecule analysis: The brightness of the different mutants was assessed using single molecule spectroscopy, after expression in our cell-free system. The samples were diluted lOx in buffer for measurement. The average signal of the GFP was used to validate the correct expression of the proteins, and all mutants expressed at very similar levels, ensuring that there is no potential bias of higher polymerisation for higher protein yields. In these measurements, millions of proteins are interrogated as they diffuse in and out of the focal volume (at any given time, there are approx. 10 proteins in the focal volume and they exchange within 10 ms; we collect data for a few minutes per sample).

SFX data- 4.3 mg of MyD88TIR mixed with 0.17 mg MALTIR was used to produce 7.5 x10^8 crystals/ml and a total of 3.2 ml of crystal solution was used to collect the data set.

MicroED data - 3 EM grids were prepared, each using 3 ul of 1:50 MyD88TIR:MALTIR crystal solution.

Data exclusions

SFX hit-finding algorithm, Cheetah, was used to exclude data that did not contain Bragg peaks. Peakfinder8 was used to exclude poor diffraction images using the following parameters; minPeaks=15, minSNR=6 , threshold=400, minPix=1.

Replication

The flow-cytometry based NF-KB and clustering assays (Figure 6A and B and Supplementary Figure 5C,E-F) were performed in duplicate with reproducible results. No experiments were excluded.

The single molecule experiments (Figure 6C) were performed in triplicate or duplicate (two technical repeats per experiment) with reproducible results. No experiments were excluded. All attempts at replication were successful. TFor analysis of number of assemblies, the proteins were expressed and diluted to the exact same average GFP value. This enables a direct comparison of the raw fluorescent traces and a calculation of the number and size of MyD88 assemblies; gating was performed with a threshold of 4000 photons/ms to calculate the fraction of events corresponding to large polymers.

Time-lapse imaging of MyD88TIR microcrystal formation (Figure 7a, Supplementary Figure 10) were repeated 5 times and each experiment showed similar results.

SFX data- The experiment was a result of 12 hours of beam time at the LCLS. 1,029,868 diffraction patterns were collected during beam time, 13,528 were identified as hits and 4,725 were indexed. The robustness of the data reduction was tested by assessing crystallography criteria such as CC*, CC1/2, Rsplit, mulitplicity, I/sigma etc which are well established methods.

MicroED data - 3 EM grids (3ul crystal solution each) were prepared and data were collected in three 4 hour sessions on a JEOL 2100 LaB6 TEM. Data were collected of 30 crystals, 18 crystals were selected for data merging based on crystallographic criteria such as Pearson CC, CC1/2, I/sig(I), Rmerge etc.

Randomization

This study used cell populations, not patients or animals. Splitting a large population of cells into treatment groups is an inherent randomisation process.

SFX data- individual microcrystals were randomly orientated in the X-ray beam as a result of the sample injection process. The crystals tumble through the X-ray beam and at any given point will be exposed to X-rays. Therefore each diffraction pattern is collected in a random orientation and the CrystFel program was used to assembled data set.

MicroED data - data were collected of crystals in random orientation, crystals were not aligned on a major zone axis prior to data collection

Blinding	Analysis was not blinded. The analysis of quantitative data was performed by a standardised procedure, the same for every group, and blinding would not alter any results.<br><br>SFX data- not relevant to this macrocrystalllography study.<br><br>MicroED data- not relevant to this macrocrystalllography study.

Reporting for specific materials, systems and methods

We require information from authors about some types of materials, experimental systems and methods used in many studies. Here, indicate whether each material, system or method listed is relevant to your study. If you are not sure if a list item applies to your research, read the appropriate section before selecting a response.

Materials & experimental systems

n/a	Involved in the study
☐	☒ Antibodies
☐	☒ Eukaryotic cell lines
☒	☐ Palaeontology and archaeology
☒	☐ Animals and other organisms
☒	☐ Human research participants
☒	☐ Clinical data
☒	☐ Dual use research of concern

Methods

n/a	Involved in the study
☒	☐ ChIP-seq
☐	☒ Flow cytometry
☒	☐ MRI-based neuroimaging

Antibodies

Antibodies used	Primary antibody: Anti-V5 rabbit monoclonal (D3H8Q) antibody (Cell Signaling Technology Ref no 07/2019, lot 6).<br>Secondary antibody: Goat anti-rabbit–Alexa Fluor-488 (Life Technologies A11008, Lot 140 8830)
Validation	The anti-V5 antibody recognises an epitope tag that can be fused with proteins and is originally derived from protein from simian virus 5. It is validated by the supplier by western blot, immunofluorescent staining and flow cytometry, using cells with or without transfection to express a V5-fusion protein. This is clearly shown on the Cell Signalling Technology website for Catalog #13202, where there is no antibody signal in non-expressing cells. We confirmed this validation by showing lack of staining in untransfected cells that do not express the antigen, and positive staining in cells expressing MyD88-V5 fusion protein (Supplementary Figure 5b).<br>The secondary antibodies were validated by lack of staining of cells that had not been stained with their cognate primary antigen.

Eukaryotic cell lines

Policy information about cell lines

Cell line source(s)	HEK-Blue human TLR4 (hTLR4) cell line (InvivoGen).<br>HEK-Blue-hTLR4-NF-kB-mScarlet cell line (prepared by the authors)<br>HEK-Blue-hTLR4-NF-kB-mScar-MyD88 KO cell line (prepared by the authors)
Authentication	The HEK-Blue human TLR4 cells line that we modified was prepared by Invivogen, and validated by them as a TLR4 reporter cell line. The actual identity of the background cell line, and whether it is genuinely HEK293 is not an issue, as the cell line is only used as a vehicle for the re-introduced signalling machinery. We are not studying any qualities of the background cell line per se. Consequently it is not relevant to genotype the cell line and confirm its background identity. It just needs to function appropriately in TLR4 signalling assays, and it does that. Introduction of NF-kB-driven mScarlet transgene was validated by response to LPS with increased fluorescent signal. MyD88 knockout in this line was validated by loss of TLR4 signalling and western blot for MyD88.
Mycoplasma contamination	All cells were tested and shown to be mycoplasma-free.
Commonly misidentified lines<br>(See ICLAC register)	No cell lines used appear on the register of commonly misidentified cell lines.

Flow Cytometry

Plots

Confirm that:

☒ The axis labels state the marker and fluorochrome used (e.g. CD4-FITC).

☒ The axis scales are clearly visible. Include numbers along axes only for bottom left plot of group (a 'group' is an analysis of identical markers).

☒ All plots are contour plots with outliers or pseudocolor plots.

☒ A numerical value for number of cells or percentage (with statistics) is provided.

Methodology

Sample preparation	The cultured cell lines (HEK-Blue human TLR4 (hTLR4); HEK-Blue-hTLR4-NF-B-mScarlet; HEK-Blue-TLR4-NF-B-mScar-MyD88 KO) were transfected and stained as described in materials and methods.
Instrument	BD Cytoflex S flow cytometer.
Software	FlowJo software.
Cell population abundance	Not relevant, no sorting was done.
Gating strategy	Cells were first gated on a SSC vs FSC plot, to exclude debris, and then gated to select single cells on a FSC-width vs FSC-area plot. In supplementary figure 5a, the population of cells with low MyD88 expression, which can induce mScarlet-i expression upon LPS treatment of cells expressing wild-type MyD88, is indicated with a green box. The mScarlet-i positive cells within that population is indicated with a red box and is defined as cells with expression above the level in empty vector transfected cells.

☒ Tick this box to confirm that a figure exemplifying the gating strategy is provided in the Supplementary Information.
